# Genetic Diversity of Common Bean (*Phaseolus vulgaris* L.) Germplasm Resources in Chongqing, Evidenced by Morphological Characterization

**DOI:** 10.3389/fgene.2020.00697

**Published:** 2020-07-08

**Authors:** Juechen Long, Jijun Zhang, Xiaochun Zhang, Jing Wu, Hong Chen, Ping Wang, Qiang Wang, Chengzhang Du

**Affiliations:** ^1^Institute of Specialty Crop, Chongqing Academy of Agricultural Sciences, Chongqing, China; ^2^Institute of Crop Science, Chinese Academy of Agricultural Sciences, Beijing, China

**Keywords:** common bean, genetic diversity, cluster analysis, principal component analysis, morphological characterization

## Abstract

In this study, the genetic diversity of 115 common bean germplasm resources collected from 27 counties in Chongqing over 3 years (2015–2017) was assessed. The results showed that the genetic diversity of the common bean germplasm resources was high, with an average diversity index of 1.447. The diversity of the qualitative traits of each organ was ranked as seed (*H′* = 1.40) > pod (*H′* = 1.062) > plant (*H′* = 0.64) > leaf (*H′* = 0.565), while the diversity of the quantitative traits of each organ was ranked as seed (*H′* = 2.021) > pod (*H′* = 1.989) > phenology (*H′* = 1.941) > plant (*H′* = 1.646). In the cluster analysis, 115 accessions were clustered into four groups. The accessions in the first and fourth group were characteristic of the Andean gene pool, while the accessions in the second group were characteristic of the Mesoamerican gene pool. However, the accessions of the third group possessed the combined characteristics of both gene pools and were thus classified as introgressed type. Four principal components represented 40.30% of the morphological diversity, with the first principal component representing the traits associated with plant growth; the second principal component representing flower characteristics; the third principal component being composed of seed characters; and the fourth principal component representing pod characteristics. The common bean germplasm resources are widely distributed in Chongqing, and the introgressed types are more prevalent in this region than elsewhere, with a rich genetic diversity and high utilization value in genetic breeding.

## Introduction

Common bean (*Phaseolus vulgaris* L.) is one of the most important legumes and is high in protein, low in fat, and rich in vitamins and dietary fiber. The regular consumption of common bean can reduce coronary heart disease, type II diabetes, and cancer (Kutoš et al., 2003; Krupa, 2008). Common bean is grown and produced all over the world. More than 120 countries or regions grow common bean, encompassing a total area of 28.78 million hm^2^ (FAO, 2018). The total production in China is 1.14 million tons and the cultivation area is 749,860 hm^2^ (FAO, 2018). Common bean originated in central and south America (Delgadosalinas et al., 1999, 2006; Bitocchi et al., 2012; Desiderio et al., 2013; Bellucci et al., 2014). Over the course of its evolution and domestication, common bean eventually formed two separate gene pools, namely the Mesoamerican gene pool and the Andean gene pool (Debouck and Smartt, 1995). During its global domestication (Gepts et al., 1991; Kami et al., 1995), several changes in its morphological characteristics occurred, including seed and leaf enlargement, alterations in growth habits and photoperiodic response, and changes in seed color and seed coat markings (Singh et al., 1991; Mcclean et al., 2004). The two gene pools have been divided into seven races. The Mesoamerican gene pool contains four races: Durango (D), Jalisco (J), Central America (M), and Guatemala (G), and the Andean gene pool contains three races: Chile (C), New Granda (N), and Peru (P) (Beebe et al., 2001). Common bean was introduced into Europe in the early 16th century and spread rapidly from Europe to the Middle East, West Asia, and other regions in the 16th and 17th centuries (Zheng, 1997). Ultimately, centers of origin were formed in Europe (Santalla et al., 2002; Angioi et al., 2010, 2011), Brazil (Burle et al., 2010), South Africa (Martin and Adams, 1987a, b; Asfaw et al., 2009; Blair et al., 2010), and China (Zhang et al., 2008). Common bean was introduced in China more than 400 years ago (Zheng, 1997), and long periods of domestication and artificial selection have led to an increase in its genetic diversity.

Chongqing is located in the middle and upper reaches of the Yangtze River in southwest China and the eastern edge of the Szechwan Basin. The terrain of Chongqing is mainly mountainous and hilly, with a large elevation range, a subtropical monsoon humid climate, and an obvious three-dimensional climate. It has been divided into four natural ecological regions (Luo et al., 2006). In Chongqing, the tender bean pods of common bean are stir-fried, stewed, and boiled, while the seeds are typically cooked in soup. The people from the mountainous areas often stew potatoes with the tender pods as their staple food. The mountainous areas of Chongqing are inhabited by local minorities such as tujia, miao, yi, and gelao (Chongqing Ethnic and Religious Affairs Commission, 2002). The common bean germplasm resources also differ between populations from different ethnic cultural backgrounds. Common bean germplasm resources have been selected and domesticated according to the specific production and life activities of people, resulting in the formation of a unique distribution structure of common bean germplasm resources in Chongqing. In this study, 115 common bean germplasm resources were collected from 27 counties in Chongqing over 3 years (2015–2017). Their distribution was investigated and genetic diversity analysis of the agronomic traits was carried out in order to clarify the distribution structure and genetic diversity of common bean in Chongqing, providing support for the development of excellent germplasm and genetic resources.

## Materials and Methods

### Plant Materials

**FIGURE 1:**
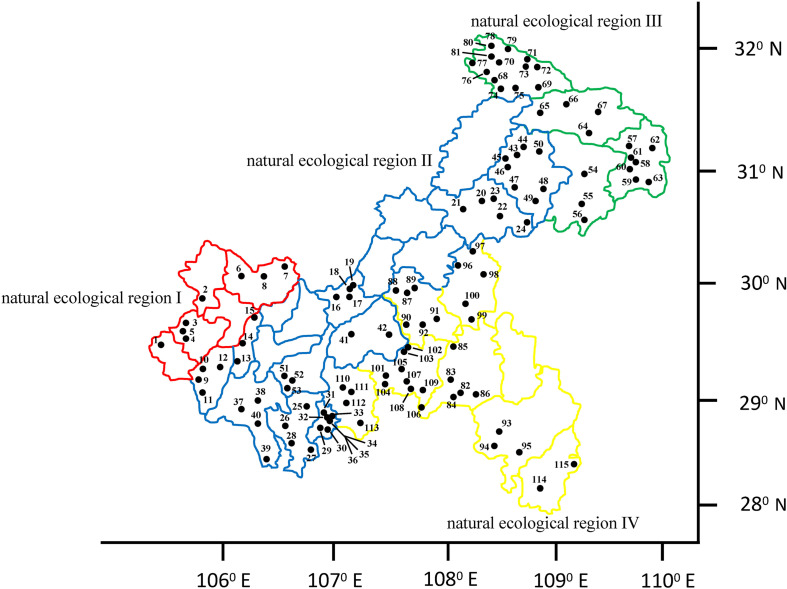
Distribution of the common bean germplasm resources in the four natural ecological regions of Chongqing.

Based on the third national survey of crop germplasm resources, this study investigated the common bean germplasm resources in different natural ecological regions of Chongqing from 2015 to 2017. A total of 115 common bean germplasm resources were collected, all of which originated from four natural ecological regions in Chongqing (natural ecological region I, Szechwan Basin agricultural natural ecological region; natural ecological region II, Three Gorges region and the parallel ridge valley compound natural ecological region; natural ecological region III, Qinling-Daba Mountain evergreen, broadleaf, and deciduous forest natural ecological region; natural ecological region IV, Southeast Chongqing montane evergreen broadleaf forest natural ecological region) (Luo et al., 2006). There were eight accessions distributed in natural ecological region I, 45 accessions in natural ecological region II, 28 accessions in natural ecological region III, and 34 accessions in natural ecological region IV (Figure 1).

### Field Experiments and Phenotyping

Agronomic traits were investigated at the experimental base in Wu long (29.52°N, 107.65°E) at 1280 m above sea level. The average annual temperature was 14.8°C in 2017 and 2018, and the soil is sandy loam with good drainage. The experimental design was a randomized complete block with three repetitions. The experimental plots consisted of six rows with a planting distance of 0.75 m between the rows and 0.25 m within the rows, and with two seeds sown per planting station. The agronomic practices were the same for all plots and are typically used by most farmers.

Twenty-nine morpho-agronomic characters were measured for the 115 accessions based on the Descriptors and Date Standard for Common Bean (*Phaseolus vulgaris* L.) (Wang et al., 2006). Hypocotyl pigmentation (HP), color of standard (CS), color of wings (CW), growth habit (GH), leaf shape (LS), podding habit (PHA), stem type (ST), and phenological characters were recorded in the field. Pod color (PC), plant height (PH), node number of the main stem (NS), number of the primary branches (NB), number of pods per plant (NP), shape of the pod (SP), shape of the pod apex (SPA), pod surface (PS), pod length (PL), pod width (PW), number of seeds per pod (NSP), seed shape (SS), seed coat color (SCC), speckle of the seed coat (SSC), color speckle of the seed coat (CSSC), hilum color (HC), seed length (SL), seed width (SW), hundred-seed weight (HSW), and length/width of the seed (LWS) were recorded at harvest. There were 16 qualitative traits and 13 quantitative traits. All the traits evaluated are listed in Tables 1, 2.

**TABLE 1:** Variable sets and 13 observed common bean quantitative characters, codes, measurement units, and measurement procedures.

**Variable set (plant organ)**	**Character**	**Code**	**Measurement unit and measurement/sampling procedure**
Phenology	Days to flowering	DF	No. of days from emergence to the time that 80% of plants within the center rows were flowering
	Days to physiological maturity	DM	No. of days from sowing to physiological maturity (80% of pods dry within the two center rows)
Plant	Plant height	PH	cm—measured at harvest; 10 plants within plot center
	Node number of the main stem	NS	No. of team section; measured at harvest; 10 plants within plot center
	Number of primary branches	NB	No. of team branches; measured at harvest; 10 plants within plot center
Pod	Number of pods per plant	NP	No. of pods per plant; measured at harvest; 10 plants within plot center
	Pod length	PL	cm—measured at harvest; 10 pods per plant from 10 plants within plot center
	Pod width	PW	cm—measured at harvest; 10 pods per plant from 10 plants within plot center
	Number of seeds per pod	NSP	Counted at harvest, for 10 pods per plant from 10 plants within plot center
Seed	Seed length	SL	cm—measured after 6 days sun drying, postharvest, 10 plants within plot center (5 pods per plant and 3 seed per pod
	Seed width	SW	cm—measured after 6 days sun drying, postharvest, 10 plants within plot center (5 pods per plant and 3 seed per pod
	Length/width of seed	LWS	Seed length - Seed width ratio
	Hundred-seed weight	HSW	g—measured at physiological maturity, on two samples of 100 seeds

**TABLE 2:** Variable sets and 16 observed common bean qualitative characters, codes, measurement units, and measurement procedures.

**Variable set (plant organ)**	**Character**	**Code**	**Measurement unit and measurement/sampling procedure**
Plant	Hypocotyl pigmentation	HP	1 = green, 2 = purple assessed at seedling stage
	Growth habit	GH	1 = erect, 2 = prostrate
	Podding habit	PHA	1 = determinate, 2 = indeterminate
	Stem type	ST	1 = normal stem, 2 = clasp stem
Leaf	Leaf shape	LS	1 = ovate, 2 = rhombic ovate
Flower	Color of standard	CS	1 = white, 2 = pinkish-white, 3 = pinkish-red, 4 = purple, 5 = light purple
	Color of wings	CW	1 = white, 2 = pinkish-white, 3 = pinkish-red, 4 = purple, 5 = light purple
Pod	Pod color	PC	1 = stripe, 2 = light brown, 3 = yellowish white, 4 = brown
	Shape of pod	SP	1 = round curved, 2 = short flat strip, 3 = long flat strip, 4 = sickle-shaped, 5 = sword-shaped, 6 = round curved stick-shaped, 7 = short round stick-shaped, 8 = long round stick-shaped
	Shape of pod apex	SPA	1 = acute apex, 2 = obtuse apex
	Pod surface	PS	1 = tiny protruding, 2 = protruding, 3 = flat
Seed	Seed shape	SS	1 = long elliptic, 2 = ovate, 3 = flat round, 4 = square, 5 = short cylinder, 6 = elliptic, 7 = kidney-shaped, 8 = round
	Seed coat color	SCC	1 = stripe, 2 = yellow, 3 = white, 4 = black, 5 = brown, 6 = yellowish-white, 7 = pinkish-red, 8 = milk white, 9 = deep yellow, 10 = red
	Speckle of seed coat	SSC	0 = free, 1 = punctiform, 2 = Stripe, 3 = reticulate
	Color speckle of seed coat	CSSC	0 = free, 1 = brown, 2 = pink, 3 = black, 4 = light brown, 5 = red
	Hilum color	HC	1 = yellowish-white, 2 = white, 3 = light brown

The 13 quantitative traits in this study included agronomic and morphological traits. All of the recording standards and methods of the traits are listed in Table 1. The 16 qualitative traits in this study included agronomic and morphological traits, and the differences among all qualitative traits were numerically assigned (Table 2).

### Phenotypic Variation Estimates

Statistical analyses were conducted using SPSS 16.0 (SPSS Inc., Chicago, IL, United States), and the average value (*X*) and standard deviation (*s*) of each trait were calculated. The coefficient of variation (*CV*) was used to evaluate the degree of phenotypic variation of each trait as follows: *CV* = *s*/*X.* Each quantitative trait of all the accessions was assigned to classes ranging from class 1: [*X*_*i*_ < (*X −* 2*s*)] to class 10: [*X*_*i*_ > (*X* + 2*s*)]. Each 0.5*s* was assigned as a class, where *X*_*i*_ was the value in class i. The Shannon–Weaver index (*H*′) was used to calculate the genetic diversity index (Khanjari et al., 2008) of each accession:

H=′-∑i=1nPilnPi

where *n* is the number of phenotypic classes for a trait, and *P*_i_ is the genotypic frequency or the proportion of the total number of entries in the *i*th class.

### Principal Component and Cluster Analyses

Principal component and cluster analysis were performed using SPSS 16.0. The spatial relationships among entries (accessions) were visualized by plotting the scores of the first and second principal components in two-dimensional space. For the cluster analysis, all the traits of each accession were standardized, and the Euclidian distances were calculated using the unweighted group average linkage method (UPGMA).

## Results

### Phenotypic Variation

According to the analysis of the 13 quantitative traits and 16 qualitative traits, the common bean germplasm resources in Chongqing are rich in genetic diversity.

**FIGURE 2:**
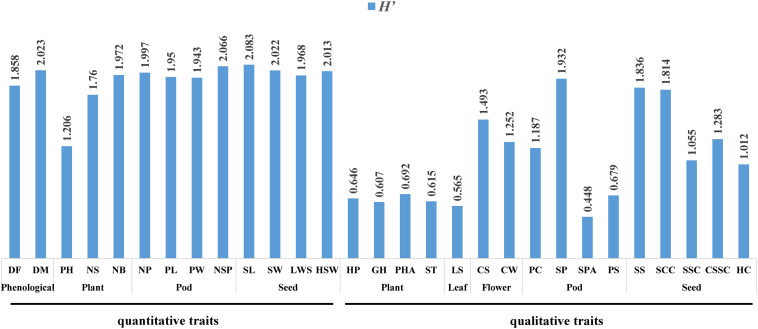
Genetic diversity of the 29 traits.

The quantitative traits are shown in Figure 2. The genetic diversity of the quantitative traits of each organ and phenological was ranked as: seed (*H′* = 2.021) > pod (*H′* = 1.989) > phenological (*H′* = 1.941) > plant (*H′* = 1.646).

For the seeds, the genetic diversity index was ranked: SL (*H′* = 2.083) > SW (*H′* = 2.022) > HSW (*H′* = 2.013) > LWS (*H′* = 1.968). The length/width of seed (LWS) of 61.7% of the accessions was at the middle level, indicating that most seeds had a more oval SS. In addition, 87% of the accessions were large to medium seeds, while only 13% were small seeds, suggesting that consumers in Chongqing prefer large and oval seeds.

For the pods, the genetic diversity index was ranked as: NSP (*H′* = 2.066) > NP (*H′* = 1.997) > PL (*H′* = 1.950) > PW (*H′* = 1.943). More than 50% of NP and NSP were medium, and the numbers at the high level were significantly higher than that at the low level, which indicated that most of the accessions have the potential to achieve high yield.

For the phenological traits, the genetic diversity index was ranked as: DM (*H′* = 2.023) > DF (*H′* = 1.858). The materials of the short growth period were 20.9%, while those of the long growth period were only 14.02%.

In terms of the plant traits, the genetic diversity index was ranked as: NB (*H′* = 1.972) > NS (*H′* = 1.760) > PH (*H′* = 1.206). Due to differences in GH, the variation coefficient of PH was the highest, while the diversity index was the lowest. In addition, more than half of the accessions were prostrate.

The qualitative traits are shown in Figure 2, where the characteristic frequency of each trait was counted and the genetic diversity coefficient was calculated. The genetic diversity of the qualitative traits of each organ was ranked as: seed (*H′* = 1.40) > pod (*H′* = 1.062) > plant (*H′* = 0.64) > leaf (*H′* = 0.565).

In terms of the plant traits, the order of the genetic diversity index was ranked as: PHA (*H′* = 0.692) > HP (*H′* = 0.646) > ST (*H′* = 0.615) > GH (*H′* = 0.607). The HP was mainly green, accounting for 65.2% of the accessions, while the remainder were purple. The GH was mainly prostrate type. There were more determinate pods than indeterminate pods in PHA, which indicated that farmers in Chongqing have a preference for prostrate-type common bean.

The LS of the accessions was mainly oval, and its genetic diversity index was only 0.565, which was the lowest of all the traits.

For the flower traits, the genetic diversity index was ranked as follows: CS (*H′* = 1.493) > CW (*H′* = 1.252). Both the CS and the CW were predominantly white.

For the pod traits, the genetic diversity index was as follows: SP (*H′* = 1.932) > PC (*H′* = 1.187) > PS (*H′* = 0.679) > SPA (*H′* = 0.448). The PC was mainly light brown, whereas only six accessions were least speckled. Most accessions belonged to the short flat strip type, while only four accessions were of the long round stick-shaped type. In terms of the shape of pod apex (SPA), 83.5% had an acute apex, while the remainder had an obtuse apex. PS was mainly characterized by tiny protrusions.

The seed traits and genetic diversity were ranked as follows: SS (*H′* = 1.836) > SCC (*H′* = 1.814) > CSSC (*H′* = 1.283) > SSC (*H′* = 1.055) > HC (*H′* = 1.012). The SS of most accessions was kidney-shaped, with 29 accessions. The majority of accessions (50) had a striped SCC, which indicated that the farmers in Chongqing have a preference for both the striped seed coats and large seeds.

### Cluster Analysis

**FIGURE 3:**
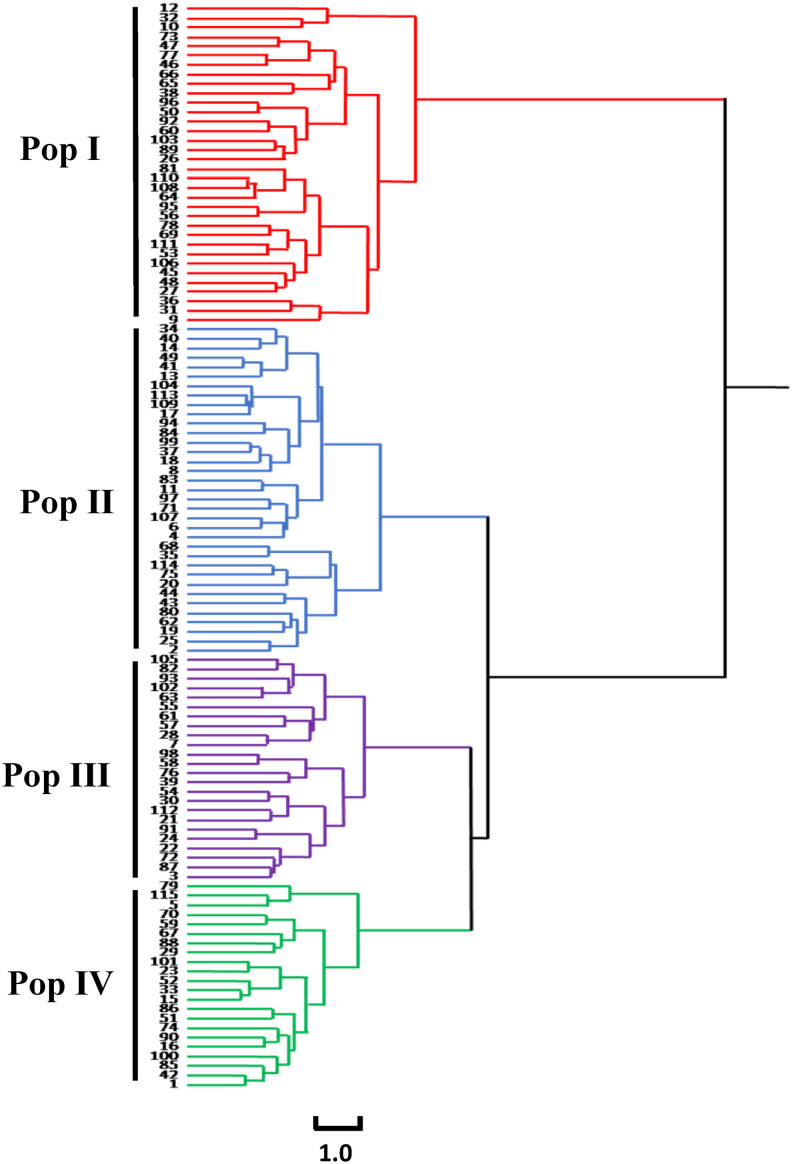
Cluster diagram of 115 common bean germplasm resources in Chongqing based on phenotypic traits.

The results of the cluster analysis showed that the 115 accessions could be clustered into four groups (Figure 3).

The first group (Pop I) contained 34 accessions, and the agronomic characteristics were as follows: the DM was 70.2 days, which was the shortest of the four groups. The HP was mainly green. The GH of the accessions in Pop I was erect. The ST was mainly normal, and the PHA was mainly determinate, with a few indeterminate accessions. The CS was predominantly light purple, while the CW was mainly white. The NP was the lowest among the four groups (7.32 pods). The SP was mainly short flat strips, while the SS was largely long ellipse. Most of the seeds had punctiform pink stripes. The LWS was the largest, indicating that the seeds of Pop I were the most elongated among the four groups. The HSW was 38.66 g.

The second group (Pop II) contained 35 accessions, and the agronomic characteristics were as follows: the DM was the longest among the four groups at 79.24 days. The HP was mainly green, and the GH of the accessions was prostrate. The PHA of the accessions was mainly indeterminate, with a few that were determinate. The CS and CW were mainly light purple. The NP was the highest among the four groups, with 10.34 pods. The pods of most of the accessions were sickle-shaped, while SS was mainly kidney-shaped. There were no stripes on the seeds of the accessions in Pop II, and the SCC was mainly white. The HSW was 34.87 g.

The third group (Pop III) contained 24 accessions, and the main agronomic traits were as follows: the DM was 77.6 days. The HP was mainly purple, and the GH of the accessions in this group was prostrate. The PHA of the accessions was mainly indeterminate. The CS and CW were mainly light purple and purple. The LS of Pop III was mainly rhombic ovate, which is unique among the four groups. The SP was mainly round curved stick-shaped, and the SS was mainly kidney-shaped, and most seeds had punctiform black stripes. The HSW was 33.03 g.

The fourth group (Pop IV) contained 22 accessions, and the main agronomic traits were as follows: the DM was 78.64 days. The GH of the accessions in Pop IV was mainly prostrate, and the PHA of the accessions was mainly indeterminate, with a few that were determinate. The CS and CW were mainly pink. The SP was mainly short flat strip with oval seeds, and most of the seeds possessed red punctiform speckles. The HSW was 40.38 g, and the seeds of Pop IV were the largest and roundest of the four groups.

The above results indicate that the characteristics of these four groups are quite different. The accessions of Pop I were more similar to the germplasm resources from the Andean gene pool due to the erect GH, white flowers, pink speckled seed coats, and large seeds. The accessions of Pop IV were more similar to the germplasm resources from the Peru (P) race in the Andean gene pool, due to the prostrate GH and larger and oval seeds. We thus classified the accessions of Pop I and Pop IV as being derived from the germplasm resources of the Andean gene pool. The accessions of Pop II were more similar to the germplasm resources of the Mesoamerican gene pool, as the seeds were small and free of speckles, and the seed coat was black, yellow, and white. Therefore, the accessions from Pop II were identified as being derived from the germplasm resources of the Mesoamerican gene pool. The accessions of Pop III were mainly prostrate, the seed coat was striped and medium in size, the flowers were mostly colorful, and there was both an indeterminate and determinate PHA, thus combining the characteristics of the Andean gene pool and the Mesoamerican gene bank. Therefore, Pop III was classified as introgressed.

### Principal Component Analysis

The results of the Principal component analysis (PCA) (Table 3) of the 29 traits of the 115 accessions indicated that the first four principal components explained 40.298% of the total variation. The first component was highly correlated with PH, HP, ST, and NS, thus representing the traits associated with plant growth. The second component was highly correlated with CS and CW, suggesting that the second principal component was related to floral traits. The third component was highly correlated with SCC, SSC, and CSSC, thus representing traits related to the seeds. The fourth principal component was composed of PL and NSP, thus representing traits related to the pods.

**TABLE 3:** The first four principal component roots and eigenvectors.

**Variable set (plant organ)**	**Trait**	**PC1**	**PC2**	**PC3**	**PC4**
Phenology	DF	0.2288	0.0015	0.0489	–0.0901
	DM	0.1813	0.0402	–0.1016	–0.3027
Plant	PH	0.3856	0.0785	–0.0465	0.1488
	HP	0.0487	0.2300	0.1761	–0.1595
	GH	–0.3884	–0.1576	–0.0562	0.1821
	ST	0.3934	0.1693	0.0152	–0.1798
	NS	0.3683	0.0905	–0.0550	0.1530
	NB	–0.1939	0.0013	0.0171	–0.3114
	PHA	0.2605	0.1355	–0.0051	–0.2478
Flower	CS	–0.0668	0.4828	0.1771	0.1306
	CW	–0.0823	0.4885	0.1895	0.1302
Leaf	LS	–0.0954	0.2202	0.0575	0.2209
Pod	PC	–0.0207	–0.0073	–0.0670	–0.0506
	NP	0.1405	–0.0024	0.1206	0.0830
	SP	–0.1296	–0.0259	–0.0926	0.0961
	SPA	0.0572	–0.0552	–0.0327	–0.1491
	PS	–0.0024	0.2159	0.1124	0.2084
	PL	0.1728	–0.1718	0.0714	0.4245
	PW	0.1873	–0.0958	–0.3082	0.0762
	NSP	0.1492	–0.1323	0.1324	0.3396
Seed	SS	0.0772	–0.0129	0.2462	0.1154
	SCC	0.0651	–0.1833	0.4235	–0.1187
	SSC	–0.0259	0.2630	–0.4607	0.0442
	CSSC	–0.0773	0.2282	–0.4483	0.0704
	SL	–0.0312	0.1507	0.0990	–0.0934
	SW	0.1097	–0.0183	–0.0504	0.1665
	LWS	–0.1097	0.1680	0.1041	–0.2171
	HC	0.0116	–0.1048	0.0893	–0.1166
	HSW	0.1647	–0.1229	–0.1831	–0.0733
Eigen-value		4.3895	2.6272	2.5903	2.0796
Contribution rate (%)		15.1364	9.0592	8.9321	7.1709
Accumulative contribution rate (%)		15.1364	24.1956	33.1276	40.2985

**FIGURE 4:**
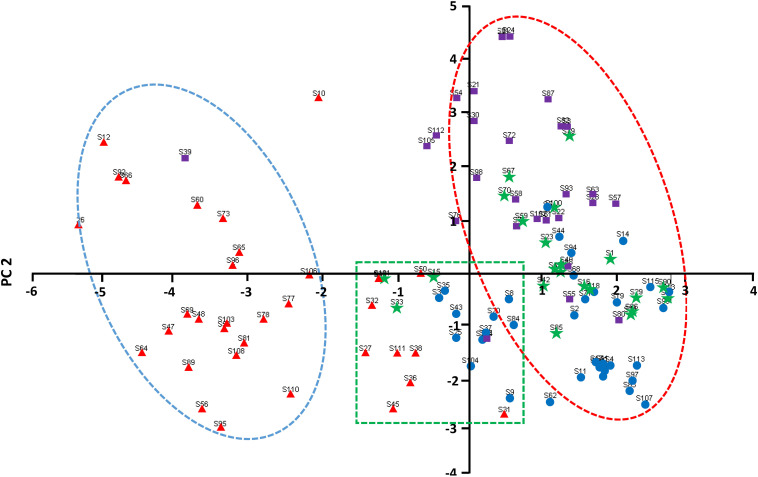
Two-dimensional PCA scatter plot of the common bean germplasm resources in Chongqing. 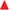
—Pop I; 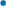
—Pop II; 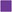
—Pop III; 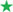
—Pop IV.

Based on the cluster analysis, the first and second principal component scores of each of the 29 phenotypic traits were used to construct a two-dimensional scatter plot (Figure 4), and the genetic structure of the common bean germplasm resources in Chongqing was further analyzed. Most of the accessions in group I were obviously concentrated on the left side of the plot, with a clear boundary with the other groups. Most of the accessions in group III were concentrated in the upper right of the plot, while most of the material in group II was concentrated in the lower right of the plot. However, the spatial distribution of the accessions in group IV was between group II and group III. In addition, there were a few outliers in the four groups.

### Geographic Distribution of the Target Germplasm

Combined with the results of the cluster analysis, there were 52 accessions derived from the Andean gene pool, which was the most widely distributed gene pool in Chongqing, accounting for 45.2%. The accessions derived from the Mesoamerican gene pool accounted for 34.8%, while the introgressed-type accessions accounted for 20%. The accessions derived from the Mesoamerican gene pool were more widely distributed than the other germplasm types in ecological region I. However, natural ecological regions II, III, and IV were the opposite, and the accessions derived from the Andean gene pool were more widely distributed. The distribution of introgressed-type accessions differed among the four ecological regions, and the proportion of introgressed-type accessions in ecological region III and IV was greater than that in the other ecological regions.

## Discussion and Conclusion

### Phenotypic Variation

The results of the diversity analysis showed that the average diversity index of the common bean germplasm resources in Chongqing was 1.447, which is higher than that in Shaanxi (*H′* = 1.344), Sichuan (*H′* = 1.390), and Yunnan (*H′* = 1.330) provinces, and lower than that in Guizhou (*H′* = 1.499) and Shanxi (*H′* = 1.450) provinces (Zhang et al., 2005).

**FIGURE 5:**
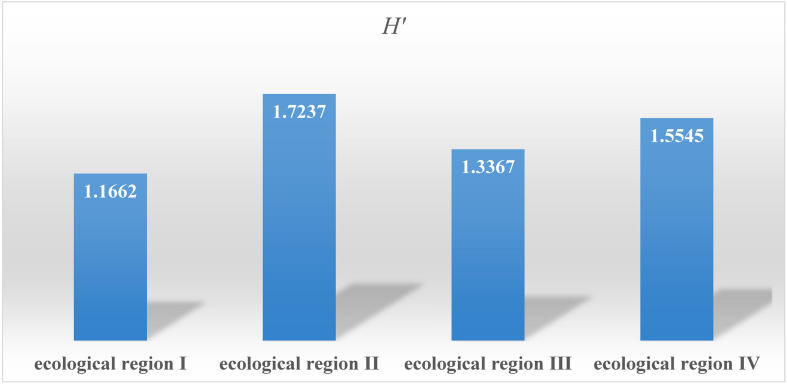
Genetic diversity analysis of accessions in each natural ecological region.

Among the four natural ecological regions in Chongqing, ecological region II has great variation in longitude, latitude, and elevation, and thus the diversity index of the accessions in this ecological zone was the highest at 1.737, followed by the accessions from ecological region IV, ecological region III, and ecological region I (Figure 5). The results of this study further supplement the diversity data of common bean germplasm resources in China.

The common bean germplasm resources in Honduras, Nicaragua, and Mexico have a short DF (García et al., 1997; Gòmez et al., 2004; Meza et al., 2013); however, 73% of the DF in the Chongqing accessions was medium, which is more similar to the common bean germplasm resources in northern Portugal (Coelho et al., 2009). Only 29.6% of the Chongqing accessions are erect in GH, which is similar to the common bean germplasm resources in Mexico, while the germplasm resources of Honduras have a more erect GH (74%) (Meza et al., 2013). The seeds of the accessions in Chongqing are mainly speckled and medium to large in size, while those in Honduras and Mexico are mainly red and small, and those in Guatemala are mainly black, bright, and small (García et al., 1997; Meza et al., 2013). This indicates that the common bean germplasm resources in Chongqing have a unique genetic diversity resulting from long-term selection and domestication.

### Cluster Analysis and Principal Component Analysis

Diversity analysis of common bean germplasm resources in Slovenia and its vicinity, the Iberian Peninsula, and central Africa has confirmed the existence of gene introgression between the two gene pools (Maras et al., 2006; Rodiño et al., 2006; Blair et al., 2010). Gene introgression also exists in the common bean germplasm resources in China (Lei, 2018). According to the cluster analysis, there were many introgressed-type accessions (20%), suggesting that introgression between the two gene pools has occurred very frequently in Chongqing. This also suggests that the farmers in Chongqing have been selecting common bean germplasm resources for a long time to suit the agricultural conditions of the region. The results of the PCA showed that the accessions could be roughly divided into three areas in the scatter plot by the first two principal components. The first principal component separated most of the erect accessions, which are derived from the Andean gene pool. The second principal component distinguished the accessions derived from Mesoamerican gene pool and the introgressed-type accessions. Notably, the scatter of the prostrate accessions in the Andean gene pool was somewhere in between, suggesting that the genetic heritage of the introgressed-type accessions was closely related to the prostrate accessions derived from the Andean gene pool.

### Geographic Distribution of the Target Germplasm

The distribution of common bean germplasm resources depends on the unique agricultural production preferences of farmers, and it has been suggested that seed selection is closely related to seed size and color (Papa and Gepts, 2003). The diversity index of seed-related traits was the highest in this study, thus confirming this view. In addition, farmers in Chongqing also tend to favor erect accessions when sowing common bean. As the terrain of the II, III, and IV natural ecological regions is dominated by steep hills and mountains, most of the common bean has been planted on slopes, and thus the farmers in these regions prefer the erect accessions in the Andean gene pool because of the reduced labor intensity. Additionally, as farmers in the mountainous areas lack means of transportation, they have been selecting and domesticating crop germplasm resources according to the specific requirements of the region for a long time, resulting in the continuous enrichment of the genetic diversity of the common bean germplasm resources. Therefore, the most introgressed-type accessions were distributed in natural ecological region III and IV.

The genetic background of the introgressed-type accessions was between the Andean gene pool and the Mesoamerican gene pool, providing new material and a bridge between the two gene pools for breeders. This is of great significance for improving the yield, quality, and disease resistance in common bean cultivation (Zhang, 2007). Breeders should thus intensify the collection of germplasm resources in ecological regions III and IV.

## Data Availability Statement

All datasets generated for this study are included in the article/supplementary material.

## Author Contributions

CD was responsible for the study design. HC and QW performed to collect all germplasm resources. JZ, XZ, and PW analyzed the data. JW and JL contributed to the writing of the manuscript. All authors read and approved the final manuscript.

## Conflict of Interest

The authors declare that the research was conducted in the absence of any commercial or financial relationships that could be construed as a potential conflict of interest.
